# Anion Exchange Membranes Obtained from Poly(arylene ether sulfone) Block Copolymers Comprising Hydrophilic and Hydrophobic Segments

**DOI:** 10.3390/polym12020325

**Published:** 2020-02-04

**Authors:** Jun Ha Kim, Mohanraj Vinothkannan, Ae Rhan Kim, Dong Jin Yoo

**Affiliations:** 1 Department of Energy Storage/Conversion Engineering of Graduate School, Hydrogen and Fuel Cell Research Center, Jeonbuk National University, Jeonju, Jeollabuk-do 54896, Korea; dtd351351351@hanmail.net; 2 R&D Education Center for Whole Life Cycle R&D of Fuel Cell Systems, Jeonbuk National University, Jeonju, Jeollabuk-do 54896, Korea; vinothkannanram@gmail.com; 3 R&D Center for CANUTECH, Business Incubation Center and Department of Bioenvironmental Chemistry, Jeonbuk National University, Jeonju, Jeollabuk-do 54896, Korea; 4 Department of Life Science, Jeonbuk National University, Jeonju, Jeollabuk-do 54896, Korea

**Keywords:** PAES, Q-PAES, anion conductivity, alkaline stability

## Abstract

The anion exchange membrane may have different physical and chemical properties, electrochemical performance and mechanical stability depending upon the monomer structure, hydrophilicity and hydrophobic repeating unit, surface form and degree of substitution of functional groups. In current work, poly(arylene ether sulfone) (PAES) block copolymer was created and used as the main chain. After controlling the amount of NBS, the degree of bromination (DB) was changed in Br-PAES. Following that, quaternized PAES (Q-PAES) was synthesized through quaternization. Q-PAES showed a tendency of enhancing water content, expansion rate, ion exchange capacity (IEC) as the degree of substitution of functional groups increased. However, it was confirmed that tensile strength and dimensional properties of membrane reduced while swelling degree was increased. In addition, phase separation of membrane was identified by atomic force microscope (AFM) image, while ionic conductivity is greatly affected by phase separation. The Q-PAES membrane demonstrated a reasonable power output of around 64 mW/cm^2^ while employed as electrolyte in fuel cell operation.

## 1. Introduction

As environmental pollution and depletion of fossil fuels become severe problems to world, demand of renewable energy is continuously increasing. Fuel cells, which possess diversified useful advantages include high efficiency, wide range of fuel choices and low/undetectable emission of pollutants, are the conversion devices that change chemical energies into electrical energies. Fuel cells can be categorized into various sorts depending on their operating temperature or electrolyte [1]. Among them, polymer electrolyte fuel cell (PEFC) has received a lot of attention owing to its low operating temperature and high density of energy, quick start up and shutdown times [2]. Despite advantages of PEFC, commercialization is still hampered due to high cost materials i.e., perfluorinated sulfonic acid (PFSA) membrane as electrolyte and noble metals as electrocatalyst. Accordingly, various efforts are being devoted to fabricate alternative fuel cells in order to overcome these drawbacks. Alkaline fuel cell (AFC) is attracting massive attention as one of the replacements owing to the advantages that the oxygen reduction reaction (ORR), an important chemical reaction in the efficiency of fuel cells, is fast in an alkaline environment, which is causing it feasible to use non-noble metal catalysts (like Ni and Co) [3].

A large number of studies about the AFC have been conducted in the last decade and a lot of progress has been made. As the initial AFC had used potassium hydroxide (KOH) solution as a liquid electrolyte, problems containing the electrolyte leakage and the precipitation of potassium carbonate (K_2_CO_3_) have been found [4]. However, the above problems were solved with the development of solid electrolyte membrane. Various polymer matrix structures such as poly(arylene ether ketone) [5], poly(arylene ether sulfone) (PAES) [6,7], poly(phenylene oxide) [8,9] and poly(vinyl alcohol) [10,11] have been widely investigated. In addition, the stability of diversified of cationic functional groups have been the focus of many efforts, such as trimethyl amine, phosphonium, imidazolium, 1,4-diazabicyclo[2.2.2]octane (DABCO) and so on [12].

Although there is a lot of development of anion exchange membrane (AEM) as mentioned above, some factors of AEM hinder the practical application, which remains a challenge to be improved. There is a disadvantage that hydroxide (OH^‒^) ions moving through the electrolyte in AEM exhibit low ion conductivity because of low diffusion coefficient when compared to H^+^ in PEFC [13]. In addition, insufficient alkali stability at high pH conditions interferes with the practical application of AEM [14,15]. Various studies are under way to overcome these weaknesses. Even though the same materials are used, random copolymer and block copolymer which differ in the polymerization process show different physicochemical properties. A well-defined multiblock structure was responsible for interconnected ion transport channels which is indicating the outstanding hydroxide conductivity. It is very important to understand the change of properties according to the morphology of polymers.

In this work, 3,3’,5,5’-tetramethylbiphenyl-4,4’-diol which has four bromination site and bis(4-chlorophenyl) sulfone were used for hydrophilic phase. Hydrophobic phase consisted of bisphenol A and decafluorobiphenyl. From these, a well-controlled PAES block copolymer was synthesized. Various membranes were created by adjusting bromination on the synthesized block copolymer. The designed AEMs are characterized by ^1^H-NMR and FT-IR. To end, we selected a bicyclic diamine, DABCO, as the diamine to production of novel quaternary ammonium (QA) groups because of its greater structural stability while compared to stabilities of alternate trimethyl ammonium groups.

## 2. Experimental

### 2.1. Materials

3,3′-Tetramethylbiphenyl-4,4′-diol (TMBD, >98%) and decafluorobiphenyl (DFBP, >98%) were purchased from Tokyo Chemical Industries (Tokyo, Japan). *N*-bromosuccinimide (NBS, 99%), bis(4-chlorophenyl) sulfone (BCPS, 98%), anhydrous *N*,*N*-dimethylacetamide (DMAc, 99.8%) and anhydrous toluene were procured from Sigma-Aldrich (Seoul, South Korea). 1,4-DABCO, 98% and bisphenol A was purchased from Alfa Aesar (Seoul, South Korea). Benzoyl peroxide (BPO, >97%), K_2_CO_3_, 99.5%, 1,1,2,2-tetrachloroethane (TCE), methanol, ethanol and acetone were acquired from Daejung reagents (Siheung, South Korea).

### 2.2. Synthesis of Quaternized PAES (Q-PAES) Membranes

#### 2.2.1. Synthesis of Hydrophilic Precursors

PAES block copolymer was synthesized as follows. TMBD (9.93 mmol), BCPS (9.03 mmol) and K_2_CO_3_ were mixed in a solution which contains 15 mL of toluene and 35 mL of DMAc. The reaction mixture was stirred for 2 h at 120 °C under nitrogen atmosphere. Then, the temperature was raised to 150 °C and kept for 2 h in order to eliminate the toluene. The temperature was raised to 170 °C for 45 h and then cooled down to room temperature (RT). To end, solution was dispensed to 800 mL co-solvent (acetone/methanol/deionized water, 1:6:1, *v*/*v*/*v*) to recrystallization. The product was collected by filtration and washed several times with methanol and acetone. The solid was dried at 60 °C under vacuum at least 48 h.

#### 2.2.2. Synthesis of Hydrophobic Precursors

To make the hydrophobic oligomer, bisphenol A (8.39 mmol), DFBP (9.23 mmol) and K_2_CO_3_ were added in a solution that contains 15 mL of toluene and 35 mL of DMAc with a mechanical stirrer. The reaction mixture was stirred for 2 h at 120 °C under nitrogen atmosphere. And then, the solution was heated to 150 °C for remove the toluene for 2 h. The temperature was raised to 180 °C for 36 h. After that, the reaction mixture cooled to RT then was dispensed into 800 mL co-solvent (acetone/methanol/deionized water, 1:6:1, *v*/*v*/*v*). White solid was collected by filtration and washed several times with methanol and acetone. The product was dried at 60 °C under vacuum at least 48 h.

#### 2.2.3. Synthesis of PAES Block Copolymers

**Scheme 1:**
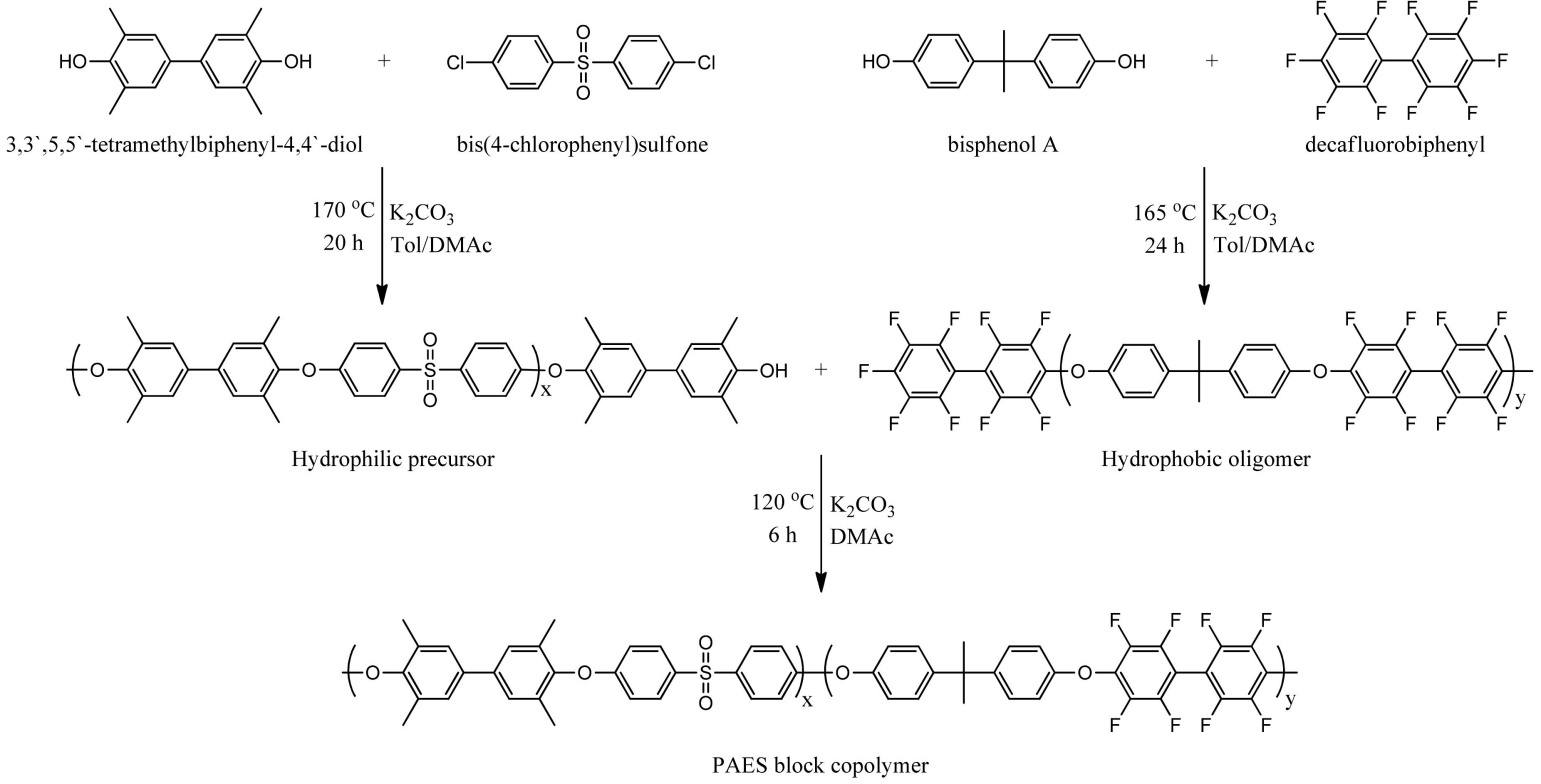
Synthesis of poly(arylene ether sulfone) (PAES) block copolymer.

The block copolymer was synthesized by condensation polymerization using hydrophilic precursor and hydrophobic oligomer. First, hydrophilic precursor (4.34 g), hydrophobic oligomer (3.94 g) and K_2_CO_3_ were added into a round bottom flask. Next, DMAc (35 mL) was added and the reaction mixture heated at 80 °C. After 6 h, the reaction mixture was cooled and recrystallized by 800 mL co-solvent (acetone/methanol/deionized water, 1:6:1, *v*/*v*/*v*). Finally, the product was collected by filtration and washed several times with methanol. Scheme 1 exhibits the pictorial expression for synthesis of PAES block copolymer.

#### 2.2.4. Bromination, Quaternization and Anionization of PAES

**Scheme 2:**
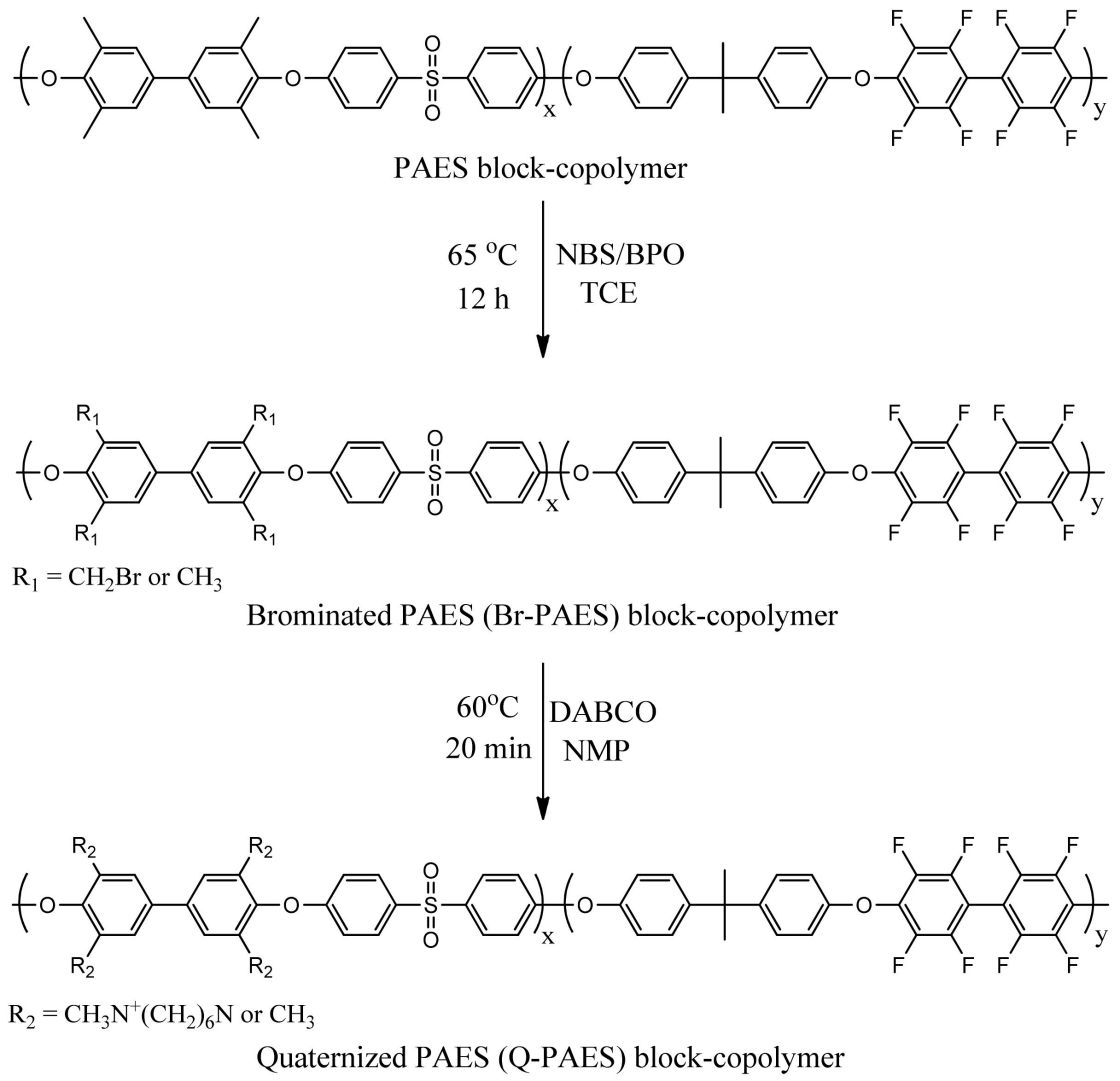
Bromination and quaternization reaction.

**Figure 1:**
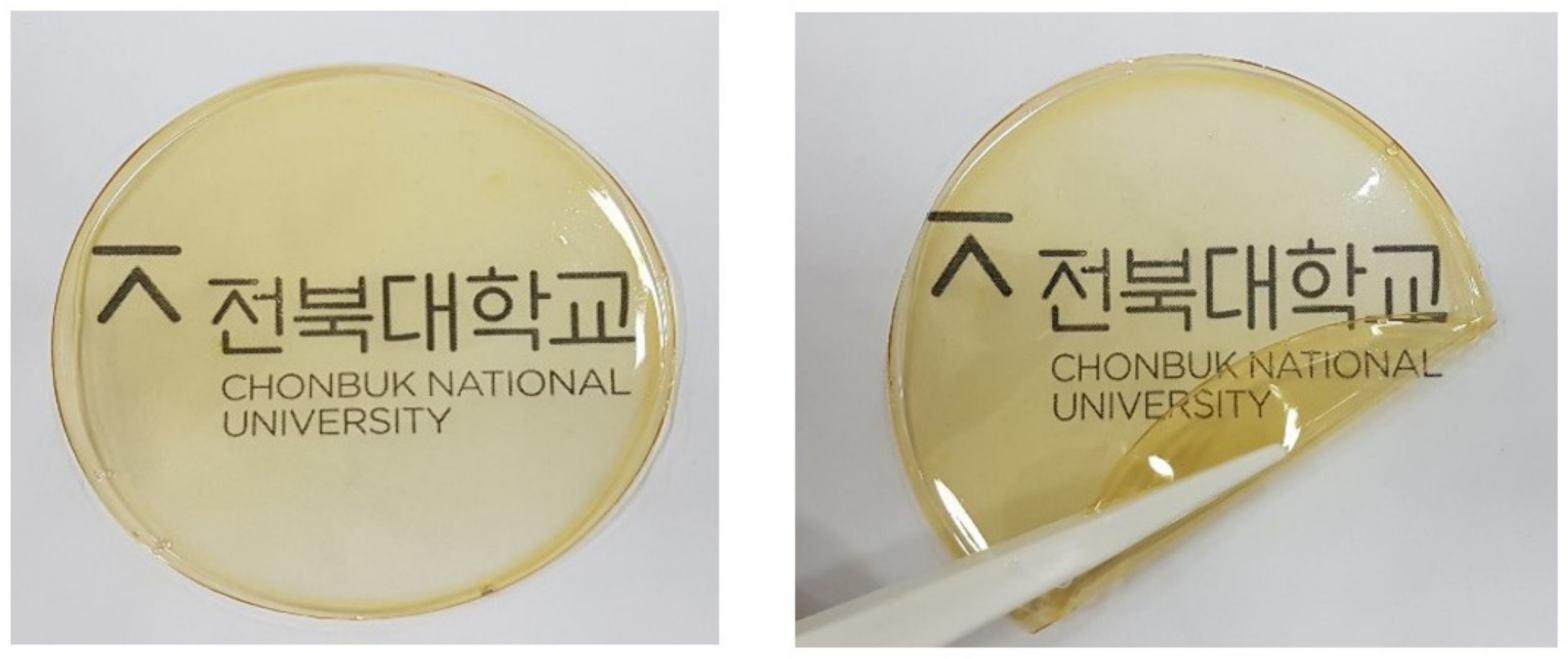
Photographs of membrane.

The bromination reaction of PAES was carried out via a free-radical substitution reaction. First, PAES block copolymers (0.8 g) was dissolved in dried 1,1’,2,2’-tetrachloroethane (35 mL) in 100 mL round bottom flaks equipped with mechanical stirrers. The reaction mixture was heated at 60 °C, then NBS and BPO were added. After 6 h, the reaction solution was cooled to RT and recrystallized with ethanol. The obtained solid was washed three times with ethanol and then dried at 60 °C under vacuum. Afterward, Q-PAES was obtained by performing the quaternization reaction of Br-PAES with DABCO. Br-PAES (0.5 g) was dissolved in NMP (5 mL) and then, in addition, DABCO was added to avoid crosslinking. Then, the reaction proceeded for 20 min at 60 °C; the reaction mixture was cast on a glass plate and dried at 70 °C under vacuum until the solvent was evaporated. The remaining DABCO and external substance were removed by immersing the fabricated membrane in methanol at RT for two days. After then the membrane was engulfed in solution of 1 M KOH for hydroxide (OH^‒^) exchange. The illustration for bromination and quaternization reaction is given in Scheme 2. Corresponding membrane photographs are shown in Figure 1.

## 3. Characterizations

Chemical structure of PAES and Br-PAES were analyzed using nuclear magnetic resonance (^1^H-NMR) spectroscopy (JNM ECA-600, 600 MHz, JEOL, Peabody, MA, USA) at the Center for University-wide Research Facilities (CURF) at Jeonbuk National University (JBNU). Substitution of functional groups by quaternization reaction was confirmed by Fourier transform infrared spectroscopy (FT-IR) Nicolet Impact 400 (Madison, WI, USA). The molecular weight (M_w_) of synthesized polymers was measured by using a gel permeation chromatography (GPC) instrument (HLP-8320, Tokyo, Japan). Thermogravimetric analysis (TGA) was performed by employing a TA instrument (Q 50, New Castle, DE, USA) with heating range of 10 °C/min in the temperature range of RT to 800 °C under nitrogen flow. Thermal stability was evaluated by utilizing differential scanning calorimetry (DSC, Q20, TA Instruments, New Castle, DE, USA) from RT to 250 °C. The temperature increase rate was set to 10 °C per minute.

Ion exchange capacity (IEC) of synthesized polymers was determined via back titration method. Q-PAES membranes in OH^‒^ form was soaked in 0.5 M HCl solution for 48 h separately. After the equilibrium, the HCl solution was titrated using 0.05 M NaOH standard solution. IEC was calculated via the given equation [16,17].

(1) $$\mathrm{IEC}\;\left(\frac{\mathrm{mequiv}}{g}\right)=\frac{C_{\mathrm{HCl}}\times V_{\mathrm{HCl}}-C_{\mathrm{NaOH}}\times V_{X,\mathrm{NaOH}}}{W_{\mathrm{dry}}}$$

where *C* means the concentration, and *V* means the volume of solution. *V*_X,NaOH_ is the volume of NaOH used for back titration.

Water uptake (WU) and swelling ratio (SR) were measured by examining weight and length difference between the dried membrane and soaked the membrane at various temperatures. After measuring the length and weight of the dried membrane, the membrane was immersed in water at appropriate temperature for 24 h and then the changes in length and weight are measured. The wet membrane was wiped quickly with a tissue and then weighed. The WU and SR were calculated using the following equation respectively. The detailed description for the following equations can be seen in literature [18,19].

(2) $$\mathrm{WU}\left(\%\right)=\frac{W_{\mathrm{wet}}-W_{\mathrm{dry}}}{W_{\mathrm{dry}}}\times 100$$

(3) $$\mathrm{SR}\left(\%\right)=\frac{L_{\mathrm{wet}}-L_{\mathrm{dry}}}{L_{\mathrm{dry}}}\times 100$$

The hydration of membrane is connected to the water content and refers to the number of water molecules absorbed per ionic group substituted in the anion exchange membrane. The membrane hydration of fabricated Q-PAES was calculated by the below mentioned relation.

(4) $$\lambda =\frac{\mathrm{WU}\left(\%\right)\times 10}{\mathrm{IEC}\times 18}$$

Atomic force microscopy (AFM) was performed to study the phase separation properties of the Q-PAES membranes. AFM examines topographical changes that occur when a tiny probe is in contact with the surface of the membranes. Nanoscope V multimode 8 AFM was employed as an analytical device for surface rendering process.

Ion conductivity is widely recognized as an essential property for evaluating the performance of a polymer electrolyte membrane because it can compare and predict how much channels are formed to transfer or conduct ions inside the membrane. For this purpose, a Bekk-Tech conductivity test cell and a PGZ 301 dynamic EIS voltammeter were used. The ion conductivity was calculated from the resistance of the membrane, and the relation is as follows.

(5) $$\sigma =\frac{L}{R\times W\times T}$$

*σ* is the ion conductivity (mS/cm), *L* is the ion cross length (cm), *R* is the resistance between the two electrodes (Ω), *W* is the width (cm), and *T* is the thickness (cm).

The chemical stability of the AEM plays a significant role in the alkaline operating environment of AFCs. The prepared Q-PAES tested for alkali stability by immersing the membrane in 1 M KOH solution at 60 °C for 1000 h, and the quantity of remaining functional groups was measured by ^1^H-NMR analysis.

## 4. Result and Discussion

### 4.1. GPC

PAES block copolymers were created by nucleophilic substitution reaction between the hydrophilic precursors and hydrophobic oligomers. The *M*_W_ of the synthesized hydrophilic precursor, hydrophobic oligomer and PAES block copolymer was measured by GPC, and results are given in Table 1. M_W_ of the synthesized PAES block copolymer was measured to be 129.9 kDa, and it was confirmed that the multiblock copolymer was successfully synthesized.

### 4.2. ^1^H-NMR

**Figure 2:**
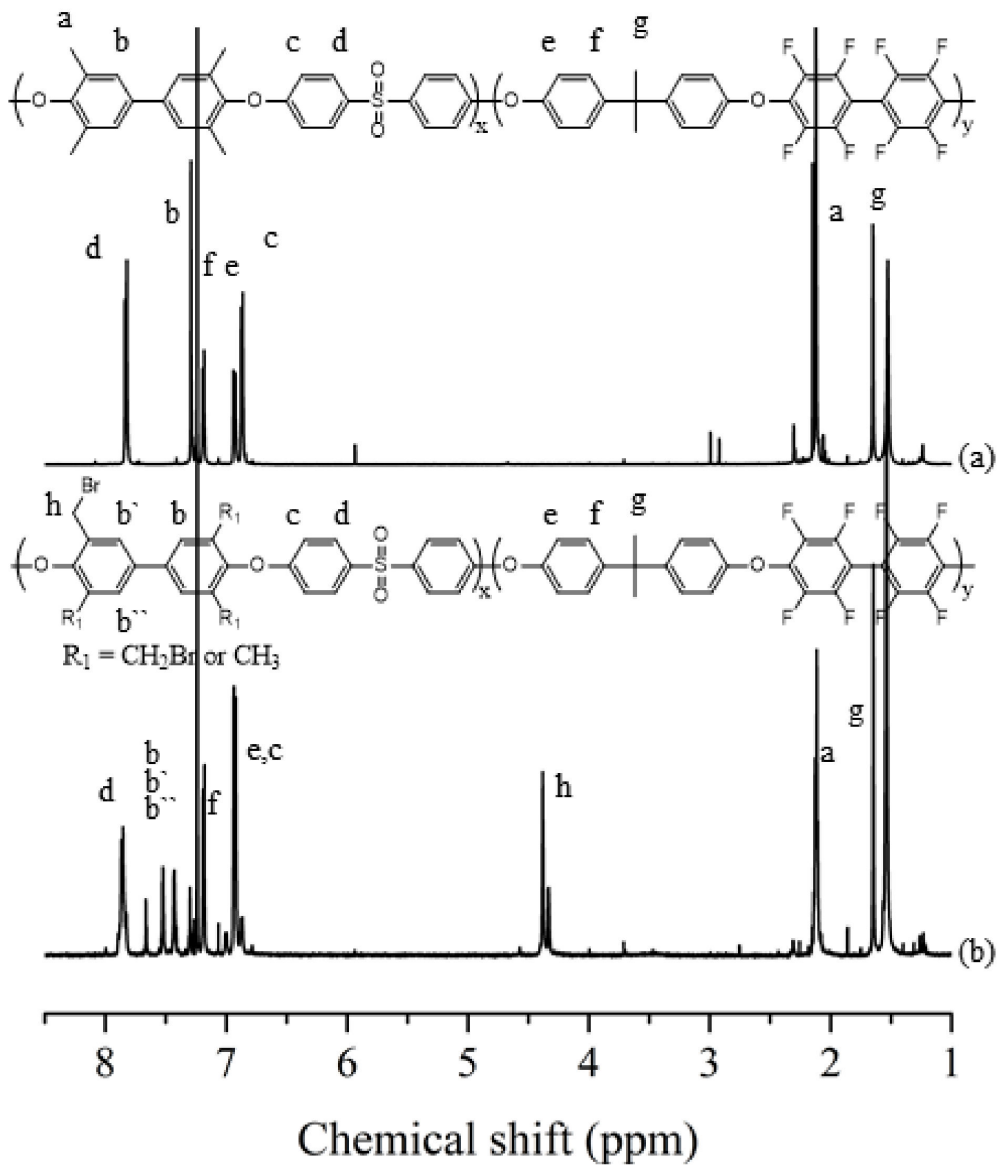
^1^H-NMR of (**a**) PAES, (**b**) Br-PAES.

After completely drying the synthesized PAES block copolymer, a bromination reaction was performed using NBS and BPO to synthesize Br-PAES. Since bromination proceeds through sensitive radical reactions, it was taken place under nitrogen atmosphere [20]. By following this procedure, various polymers with different degrees of bromination reaction were synthesized by controlling the equivalent of NBS. Afterwards, Br-PAES was dissolved in NMP solvent and subjected to quaternization using DABCO in order to obtain Q-PAES, and to introduce ionic functional groups into the polymer. Q-PAES was transferred from Br-form into OH-form in 1 M KOH for various properties and performance evaluation. In order to evaluate the structural properties of the synthesized polymer ^1^H-NMR spectrum was measured. The ^1^H-NMR results of PAES and Br-PAES in Figure 2 show that the peak *H*_a_ for the bromination reaction appears at 2.1 ppm. The aromatic protons located in the benzene ring varied between 6.8 and 7.9 ppm this is based on the observed peaks of *H*_f_, *H*_c_, and *H*_e_ at 7.2 ppm and 6.9 ppm respectively. *H*_d_, on the other hand, was found at 7.8 ppm [21,22,23]. *H*_c_ and *H*_e_ are found in the high field because the neighboring carbon is substituted by electron donor, and *H*_d_ is found in the low field due to the neighboring carbon is substituted by aliphatic carbon chains. It can be seen that ^1^H-NMR of Br-PAES shows a proton peak of a bromo methylated methyl group (–CH_2_Br) at 4.3–4.4 ppm, which was not found in PAES polymer. This proves that the bromo methylation reaction proceeded properly. The abovementioned peaks were used to calculate the degree of bromination (DB) by following the equation. The amount of NBS was adjusted to control the degree of bromo methylation, and it can be seen that the peak of 4.3–4.4 ppm increases as the amount of NBS increases. Furthermore, it also confirmed from the size of the H_a_ peak appears at 2.1 ppm accordingly.

(6) $$\mathrm{DB}\left(\%\right)=\frac{\left[A\left(H_{a}\right)-A\left(H_{a^{\prime }}\right)\right]}{A\left(H_{a}\right)}\times 100$$

**Figure 3:**
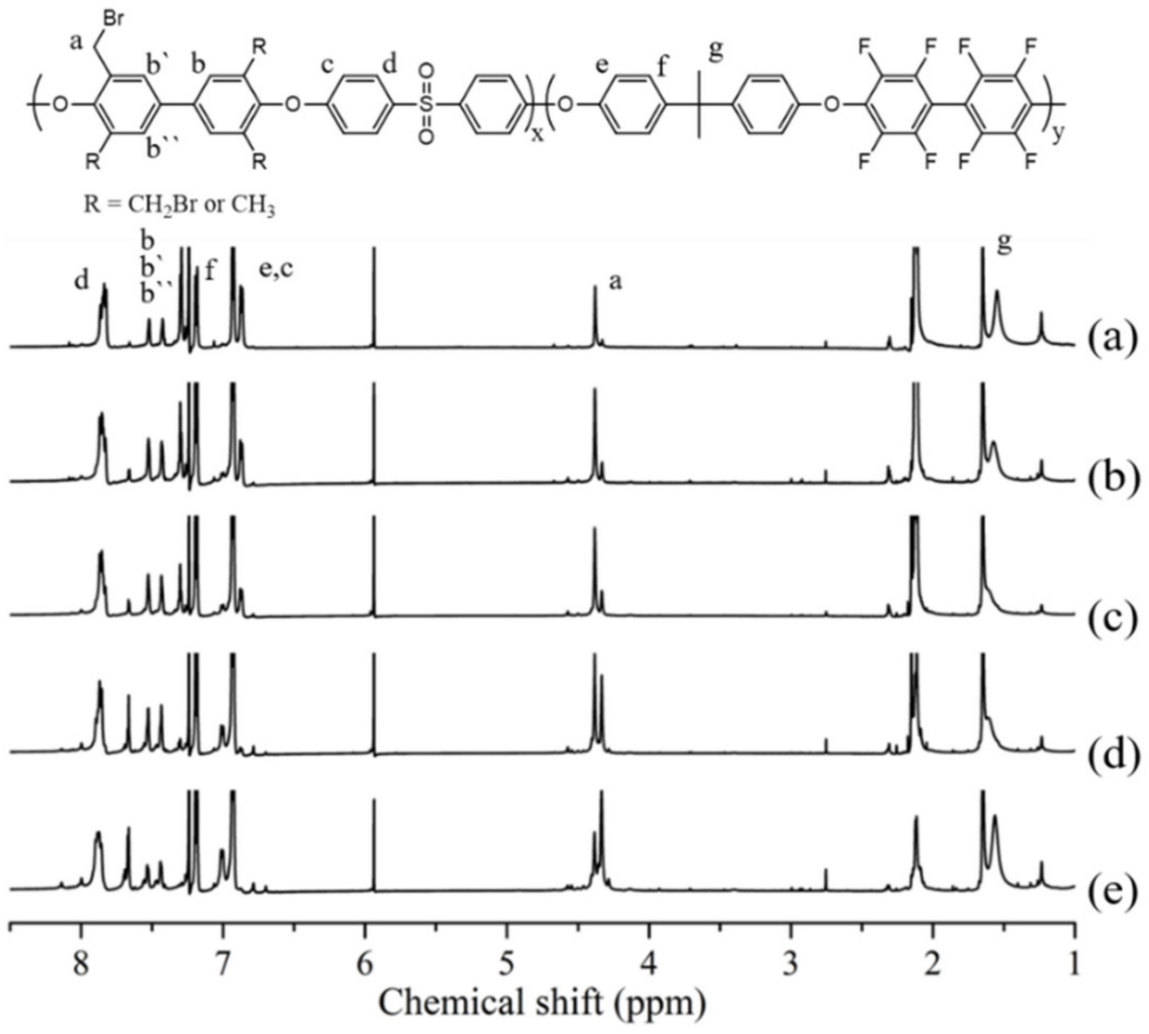
^1^H-NMR of (**a**) Br-PAES-10%, (**b**) Br-PAES-20%, (**c**) Br-PAES-30%, (**d**) Br-PAES-40%, (**e**) Br-PAES-50%.

In the above formula, A (*H*_a_) denotes the integrated area of *H*_a_ peak of PAES polymer, and A (*H*_a’_) denotes the integrated area of *H*_a_ peak of Br-PAES. Therefore, it was calculated by the area difference between proton peaks in the non-brominated polymer and the brominated polymer. As the DB of polymer increased, the peak area at 2.1 ppm became smaller, while the peak area at 4.3–4.4 ppm was increasing (Figure 3).

### 4.3. FT-IR

**Figure 4:**
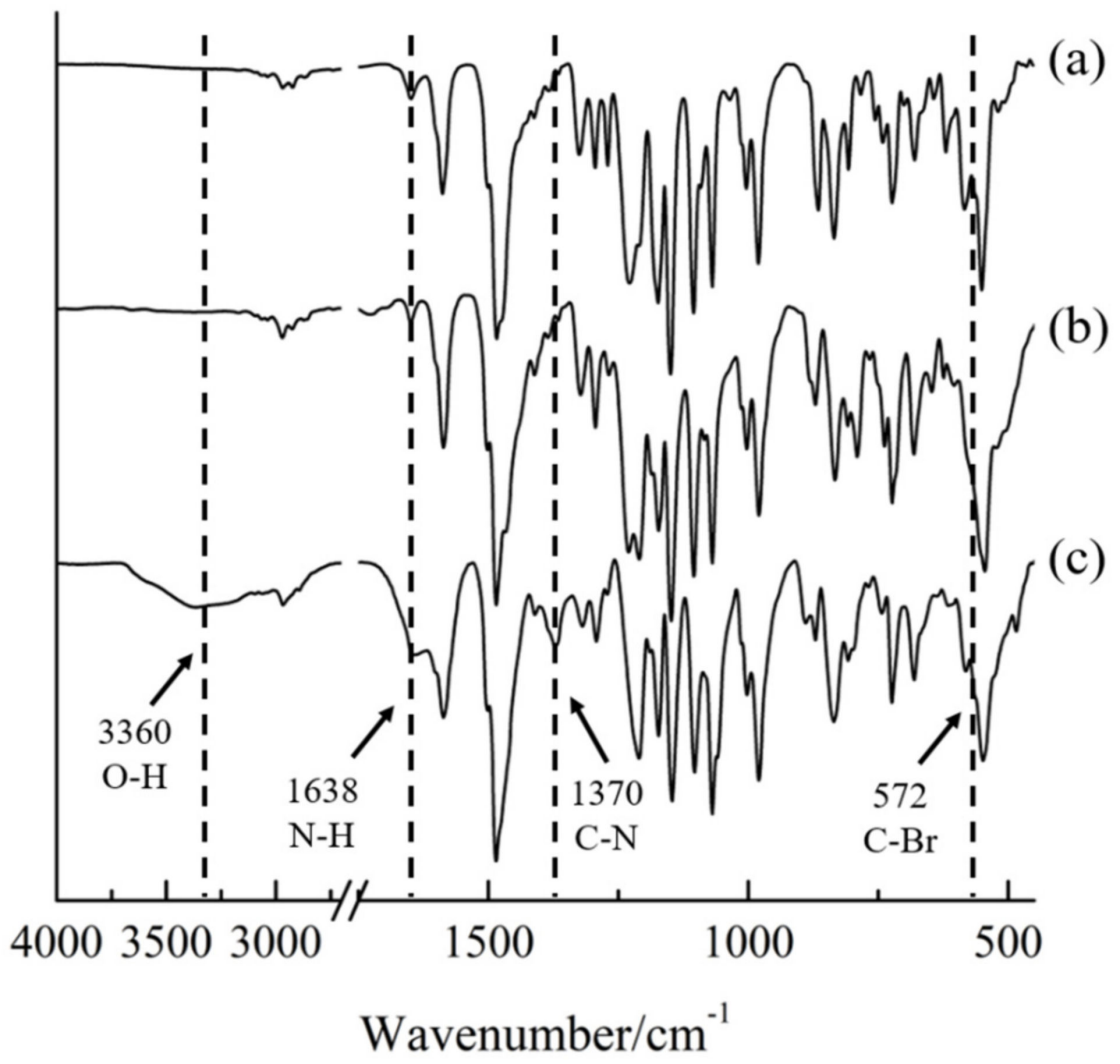
FT-IR of (**a**) PAES, (**b**) Br-PAES, (**c**) Q-PAES.

The FT-IR spectra were obtained to examine the bromination reaction and quaternization reaction of the synthesized polymer. The results are shown in Figure 4. Absorption of the peaks at 1587 and 1483 cm^‒^^1^ were common for all three polymers, PAES, Br-PAES, and Q-PAES which due to the existence of C=C bonds. In addition, the peaks of S=O and C–O bonds were found at 1154 and 1016 cm^−1^, respectively. The observed peak at 572 cm^‒1^ was due to stretching vibration of C–Br bond. The absorption peak at 572 cm^‒^^1^ was attributed to the stretching vibration of C–Br bond. In the case of Q-PAES, the presence of peaks at 3360 and 1370 cm^‒^^1^ were related to bond stretching of O–H and the C–N thus indicates the reaction proceeded successfully [24,25].

### 4.4. Solubility

Solubility of prepared PAES, Br-PAES and Q-PAES polymers was measured in order to examine their various assays. The solubility test was conducted at a concentration of 10% (*w*/*v*) at RT. All polymers are didn’t dissolved in polar protic solvents include water and methanol. However, it was found that it is dissolved in polar aprotic solvents include chloroform and DMF. Only Q-PAES was partially dissolved in DMSO. This is because the polarity is increased by the quaternary ammonium ion substituted in Q-PAES.

### 4.5. Thermal Stability

**Figure 5:**
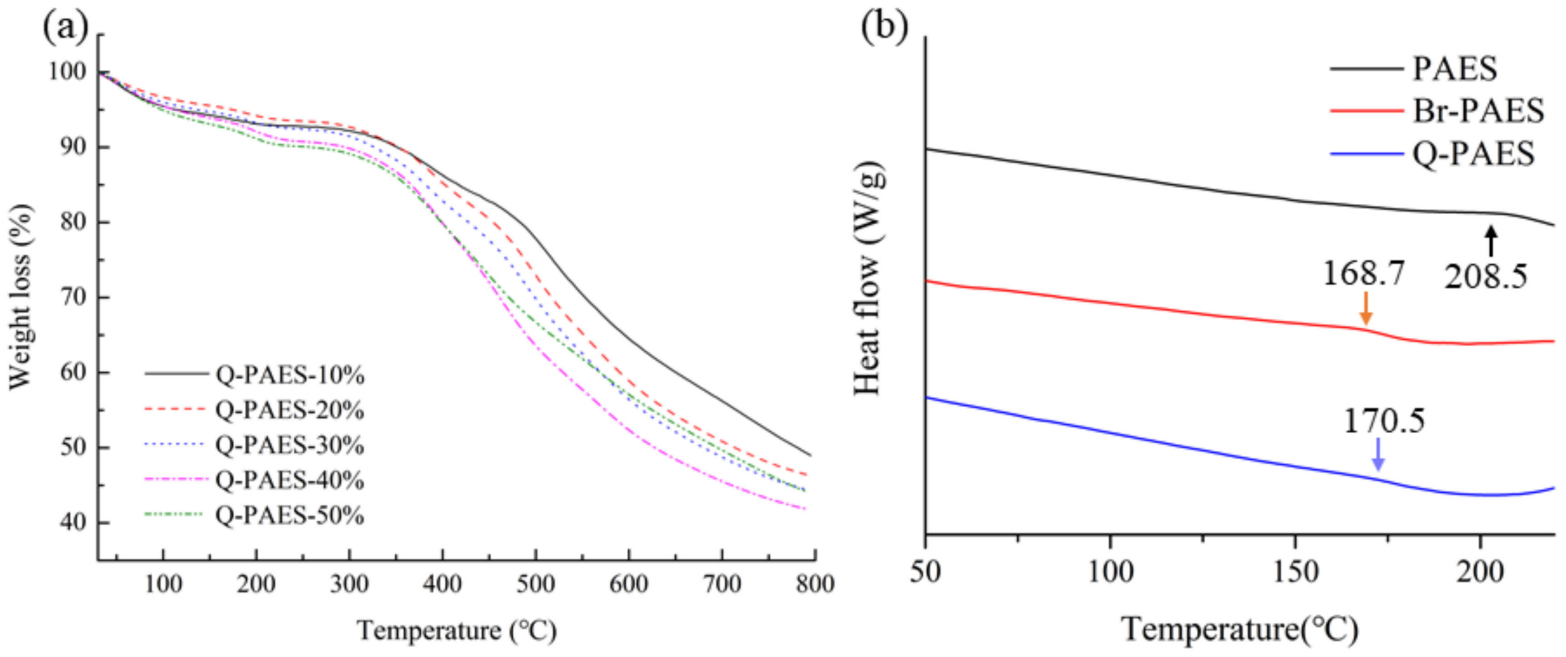
(**a**) The TGA graph and (**b**) differential scanning calorimetry (DSC) data of Q-PAESs.

TGA was measured to analyze the thermal properties of the synthesized PAES, Br-PAESs, and Q-PAESs polymers (Figure 5a). TGA analysis was measured by heating the samples from room temperature to 800 °C at the heating rate of 10 °C per minute in a nitrogen atmosphere. The TGA results of Q-PAESs showed weight loss up to 100 °C, resulting from the elimination of water molecules attached to quaternary ammonium ions. Weight loss at 180–220 °C is a result of decomposition of the quaternary ammonium group; this phenomenon became higher as the DB value increased [26].

The glass transition temperature (*T*_g_) of PAES, Br-PAES, and Q-PAES was confirmed by DSC analysis (Figure 5b). In the DSC analysis, the temperature was increased at a rate of 10 °C per minute in a nitrogen environment, and the presented DSC curves were obtained from the second scan. The *T*_g_ values of PAES, Br-PAES, and Q-PAES polymers were at 208, 168 and 170 °C, respectively. The Q-PAES is exhibited higher *T*_g_ value i.e., because of DABCO, which is bulkier than Br, was introduced into the polymer and interferes with the rotation of C–C bond in the main chain.

### 4.6. IEC, Water Uptake and Dimensional Properties

**Figure 6:**
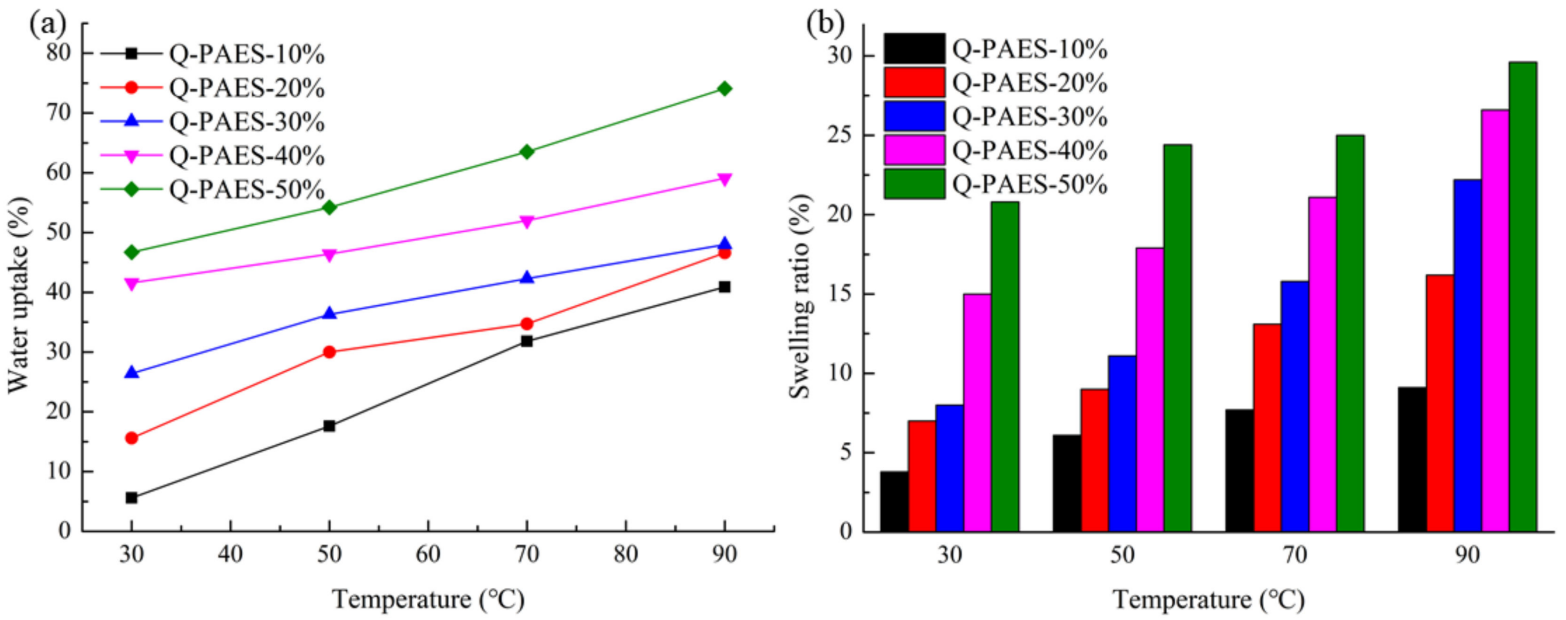
(**a**) Water uptake of fabricated membrane and (**b**) swelling ratio of Q-PAESs.

The measurement of IEC was proceeded to determine the amount of exchange of hydroxide ions per unit weight of anion exchange membrane. IEC values correlated not only with electrochemical performance but also with water moisture content, expansion rate and mechanical properties of membranes [27]. The IEC value of the synthesized AEMs was measured by following an acid-base titration technique. The measured IEC values of Q-PAES-10% and Q-PAES-50% were 1.06 and 1.87 meq/g respectively. In the AEM, the morphological change of the membrane surface occurs according to the moisture content, which may affect the IEC and the ion conductivity of the membrane. In the case of the AEM comprising of sufficient moisture content, the performance can be improved by the formation of ion channels these in turn improve the transfer of hydroxide ions. However, the water contents in the polymer matrix cause the membrane to expand in volume direction thus decline the mechanical toughness of the membrane and adversely affect its durability. Therefore, it is important to control the water uptake behavior of the membranes. The synthesized Q-PAES membrane was engulfed in 1 M KOH for 48 h, and then pretreated by dipping in 48 h in distilled water. The water content and expansion rate of various Q-PAES membranes were measured at the temperature ranges of 30, 50, 70 and 90 °C (Figure 6a). As the DB of the polymer increased, the hydrophilic part of the membrane increased, which tended to absorb water molecules rapidly. At 90 °C, Q-PAES-10% showed 40.9% water moisture content, whereas Q-PAES-50% showed higher value of 74.1%. Hydrated water also showed higher value as DB value increased. The expansion rate also followed the similar pattern to the water content rate (Figure 6b). At 90 °C, the expansion rate of Q-PAES-10% was as low as 9.1%, but the Q-PAES-50% was expanded by 29.6%, thereby confirming that the membrane stability was drastically lowered as the DB increased.

### 4.7. Morphology

**Figure 7:**
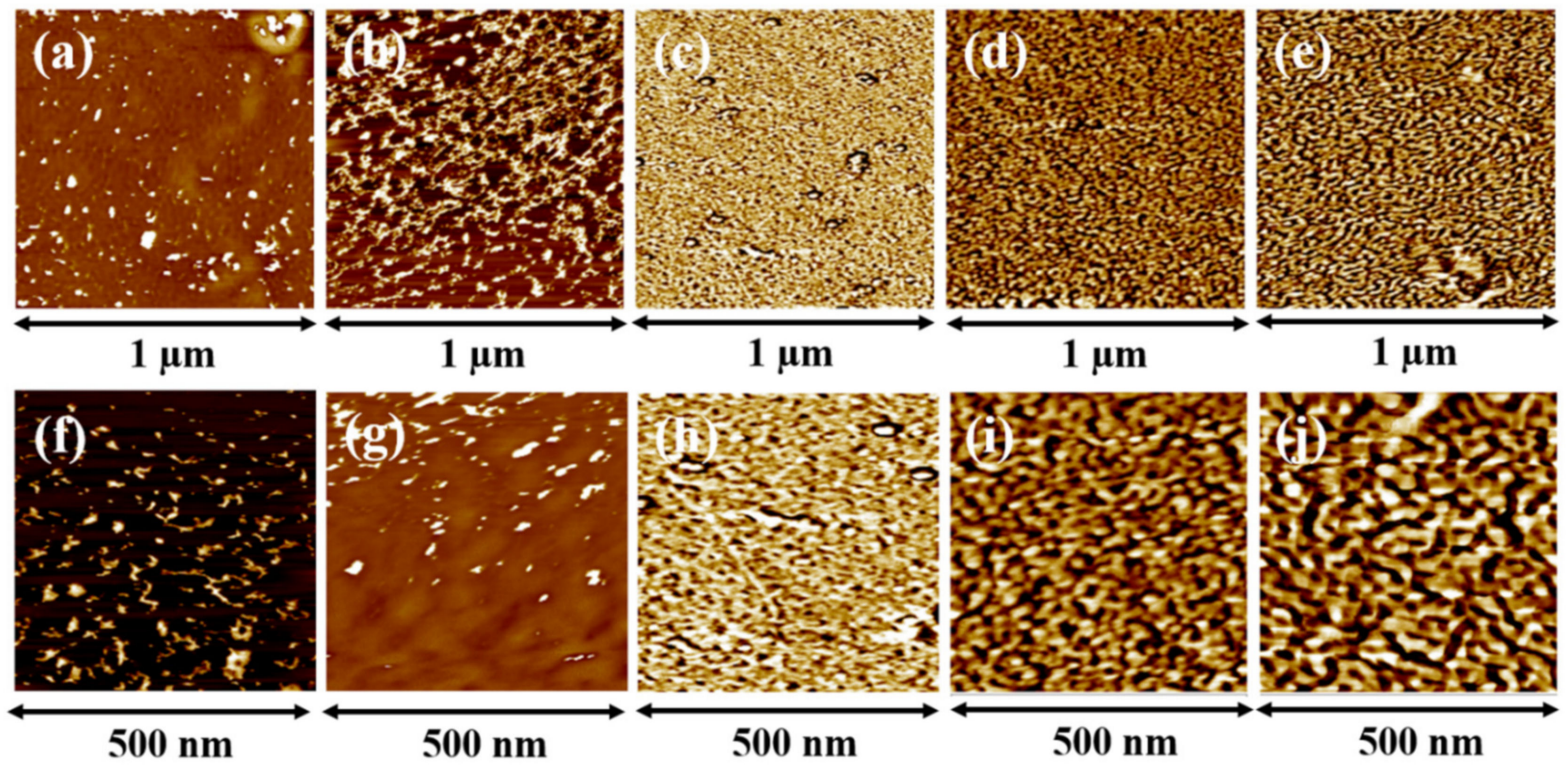
AFM phase images of (**a**,**f**) Q-PAES-10%, (**b**,**g**) Q-PAES-20%, (**c**,**h**) Q-PAES-30%, (**d**,**i**) Q-PAES-40%, (**e**,**j**) Q-PAES-50%.

The surface morphology of the AEM can impact the performance of the membrane. In particular, some researchers have reported that the formation of ion channels due to the hydrophilic and hydrophobic nano phase separation improves ion conductivity of the membrane [28]. The surface morphology of the fabricated Q-PAESs was observed via AFM, and the images are shown in Figure 7. No significant phase separation was seen in Q-PAES-10%, Q-PAES-20% and Q-PAES-30%. However, Q-PAES-40% and Q-PAES-50% possessed the nano phase separation in which bright and dark parts are clearly distinguished. Hydrophilic regions appear in darker region because the quaternary ammonium groups used as functional groups absorb water, and vice versa. As shown in the figure, as the DB value increases, the substitution of the quaternary ammonium salt also increases, so it can be seen that the nano phase separation is apparent. As revealed from Figure 7e,j, the average size of hydrophilic domains might be a 100 nm in case of Q-PAES-50% membrane.

### 4.8. Ion Conductivity and Activation Energy

**Figure 8:**
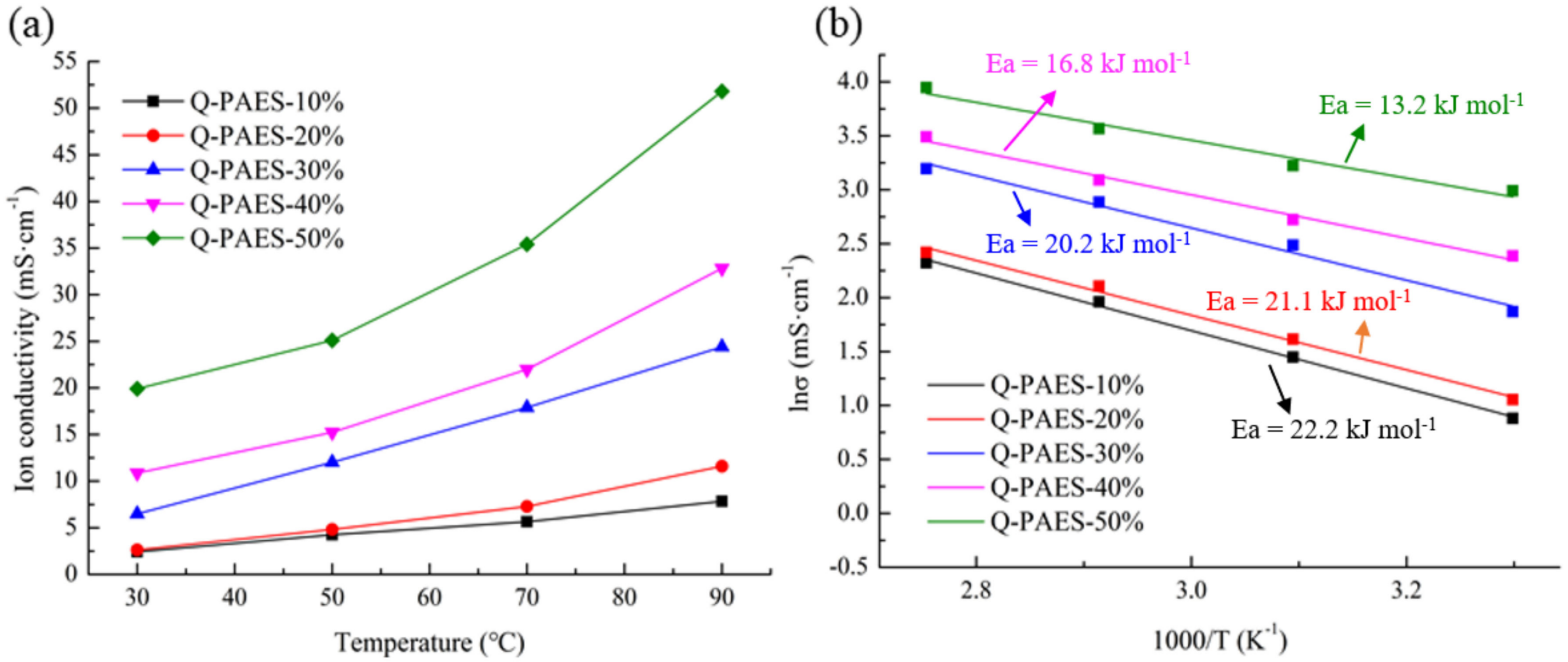
(**a**) Ion conductivity of Q-PAES membranes and (**b**) Arrhenius plot of Q-PAES membranes at diversified temperatures with 100% RH.

Ionic conductivity is a dominant factor which determine the overall performance of the anion exchange membranes. In general, ionic conductivity of membranes differs according to the several of complex factors, such as the type of polymer and the type of functional group [29,30]. Conductivity of the fabricated Q-PAES was quantified at diversified temperatures under 100% relative humidity (RH). The ionic conductivity of Q-PAES was varied due to the increased mobility of hydroxide ions when increasing the temperature. Unsurprisingly, as the DB increased, the ion conductivity value tended to increase. In particular, there is a big difference between Q-PAES-20% and Q-PAES-30%, which can be attributed to the phase separation property of Q-PAES-30% as shown in the AFM image. At 90 °C, Q-PAES-20%, Q-PAES-30%, and Q-PAES-50% were displayed ionic conductivities of 11.6, 24.4 and 51.8 mS/cm respectively (Figure 8a). On the basis of these abovementioned results, it could be concluded that the phase separation property of membrane causes the ionic conductivity to vary greatly. Arrhenius plots using ion conductivity showed that Q-PAES-50% had the highest activation value of 22.2 kJ/mol while Q-PAES-50% had the lowest value of 13.2 kJ/mol (Figure 8b).

### 4.9. Alkaline Stability and Single Cell Activity

**Figure 9:**
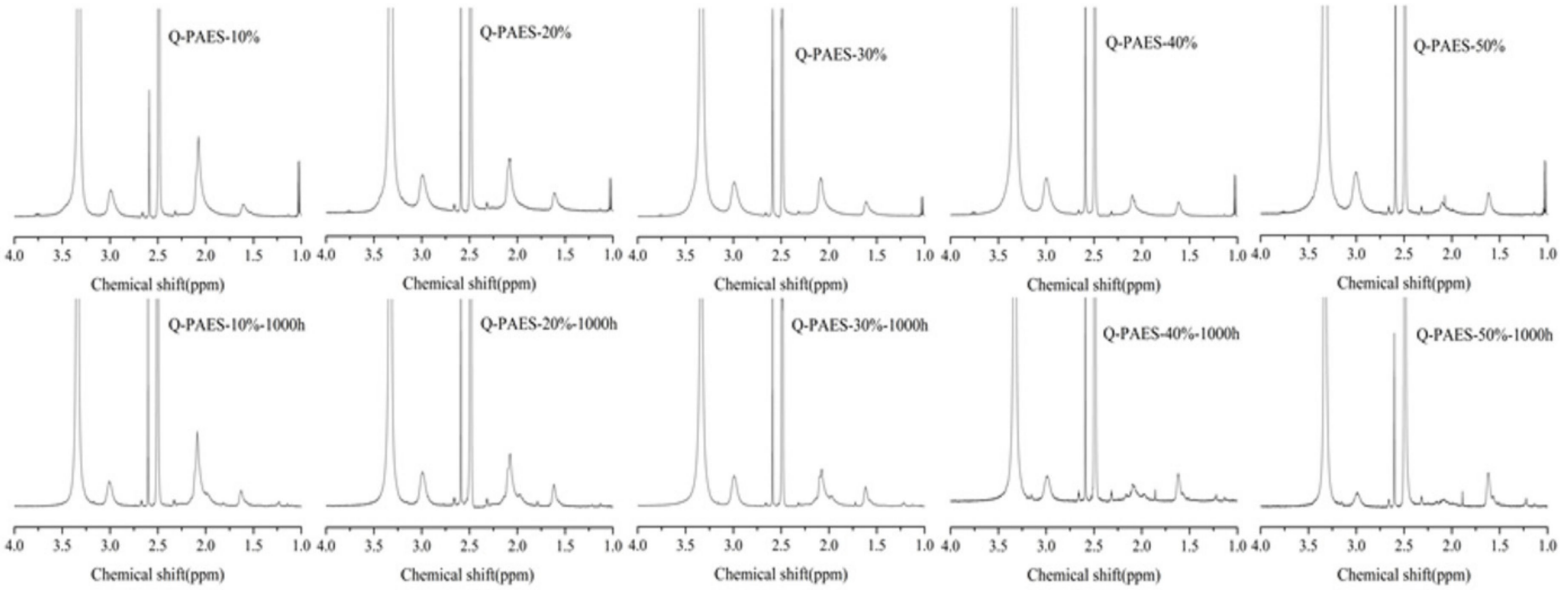
^1^H-NMR data of Q-PAES membranes before and after alkaline stability operation in 1 M KOH solution at 70 °C for 1000 h.

**Figure 10:**
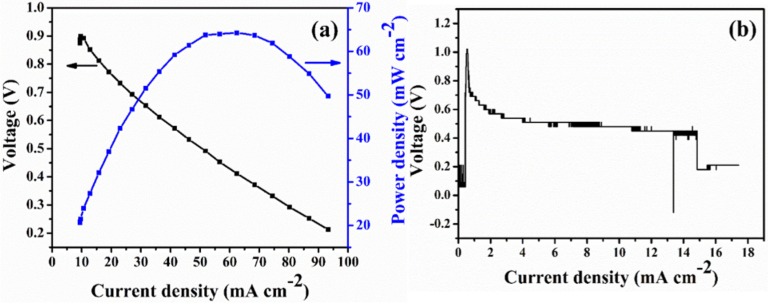
(**a**) Single fuel cell operation curve and (**b**) durability plot of Q-PAES-50% quantified at 60 °C and 100% RH. (Catalyst of anode and cathode was Pt/C, 0.3 mg/cm^2^; humidified hydrogen (100 mL) and oxygen (300 mL) were passed to anode and cathode).

The stability of the anion exchange membrane under alkaline environment is one of the problems that AFC must solve. In a typical AFC, the higher pH values of hydroxide ions under elevated operating temperature promote the decomposition of the quaternary ammonium groups in the polymer matrix which in turn higher the vulnerability of the membrane [31,32]. Alkaline stability of prepared Q-PAES membranes was examined by ^1^H-NMR analysis, after immersing the samples in 1 M KOH solution at 60 °C for 1000 h. In Figure 9, the peak measured at 3 ppm is due to the C–N binding and the area before and afterward the alkaline stability operation was compared. Nearly all functional groups remained undecomposed in Q-PAES-10%, whereas only 42% of functional groups remained in Q-PAES-50%. Therefore, as the degree of quaternization increased, it was found that the decomposition of the functional group proceeded further in alkaline environment. It can be concluded that since the functional sides of membranes act as an electron withdrawal group that lowers the electron density of the polymer main chain, as a consequence, the decomposition caused by nucleophilic hydroxide ion occurred more easily. The single cell activity of notable Q-PAES-50% was quantified. The detailed protocols for creation of membrane electrode assembly and operation of fuel cell can be observed in reference [33]. As revealed from Figure 10a, the considerable power output of 64 mW/cm^2^ was reached. Nevertheless, the Q-PAES-50% can stable be up to 18 h and lose the voltage after 18 h (Figure 10b). If additional work put forward in future, the power output and durability of membrane could be improved. According to Table 2 given, the Q-PAES-50% revealed considerable power output, but that is lower than other reported electrolytes.

## 5. Conclusions

In this study, we have successfully created AEMs with high conductivity and improved alkaline stability. Additionally, we examined the microphase separation and characteristics of polymers with different quaternization degree by controlling the DB value via controlling the amount of NBS. The synthesized polymers were analyzed by ^1^H-NMR and FT-IR to confirm the reaction was successfully carried out. Solubility measurements showed that each polymer was soluble in most polar aprotic solvents, but not in polar protic solvents such as water and methanol. In addition, only Q-PAES was partially dissolved in DMSO. This was assumed to be caused by the charged portion of Q-PAES. The thermal properties of the polymers were analyzed by TGA and DSC, and the decomposition of the functional groups was confirmed to be decomposed at about 180 °C. In the case of water moisture content and expansion rate, both tended to increase as the degree of quaternization reaction increased. Observation through the AFM image to analyze the surface of the membrane showed a marked phase separation from Q-PAES-40%. Ion conductivity of the synthesized anion exchange membrane was measured at various temperatures under 100% relative humidity. At 90 °C, Q-PAES-10% exhibited a low conductivity of 11.1 mS/cm while Q-PAES-50% possessed a higher value of 51.8 mS/cm. Alkaline stability test shows that the higher the quaternization degree, the sharper the decrease in stability under alkaline environment. This is owning to the reduction in electron density of polymer main chain which due to the substitution of functional groups, and thus cause the facile decomposition of membrane by nucleophiles. Therefore, the high degree of quaternization reaction of the anion exchange membrane may form ion channels, which may exhibit high performance. However, it is imperative to properly control the quaternization reaction of the polymer because it may affect the physical and chemical stability of the membrane.

## Figures and Tables

**Table 1:** Molecular weight and PDI of polymers.

Polymer	*M*_n_^a^ (kDa)	*M*_w_^b^ (kDa)	PDI ^c^ (*M*_w_/*M*_n_)
Hydrophilic precursor	5.5	18.3	3.3
Hydrophobic precursor	4.2	10.3	2.4
PAES block copolymer	19.2	129.9	6.7

^a^ Number average molecular weight; ^b^ weight average molecular weight; ^c^ polydispersity index.

**Table 2:** Comparison table of Q-PAES-50% with other reported membranes.

AEM ^a^	Maximum Power Output (mW/cm^2^) [RH ^b^ (%); Tem ^c^ (°C)]	Hydroxide Conductivity (mS/cm) [RH ^b^ (%); Tem ^c^ (°C)]	IEC (mmol/g)	Ref.
A-PEI-8 ^e^	-	44.2 [100; 90]	1.23	[31]
AEM-3 ^d^	152 [70; 50]	140 [100; 85]	2.89	[34]
5% IL ^f^-TnT ^g^/QAPSU ^h^	302 [100; 60]	20.8 [100; 30]	1.93	[35]
QBz ^i^-PEEK-76.0%	391 [100; 70]	155 [100; 60]	1.73	[36]
QN-PAEK ^j^/rGO ^k^ 5.0 wt %	-	116.8 [100; 90]	1.32	[18]
bQPAES-x7y32-1 ^l^	-	86.3 [100; 80]	1.15	[37]
Q-PAES-50%	64 [100; 60]	51.8 [100; 90]	1.87	current work

^a^ Anion exchange membrane; ^b^ Relative humidity; ^c^ Temperature; ^d^ Cross linked polymer membranes prepared from tetraalkylammonium-functionalized norbornene derivatives; ^e^ Alkalized poly(ether imide)s; ^f^ Ionic liquid (1-Methyl-3-(3-trimethoxysilylpropyl) imidazolium chloride); ^g^ Titanate nano tubes; ^h^ Quaternised polysulfone; ^i^ Benzylation modification of poly(ether ether ketone); ^j^ Quaternized poly(arylene ether ketone); ^k^ Reduced graphene oxide; ^l^ Benzyl-quaternary ammonium groups tethered poly(arylene ether sulfone) block copolymer ionomers.
